# The acute impact of high-dose lipid-lowering treatment on endothelial progenitor cells in patients with coronary artery disease—The REMEDY-EPC early substudy

**DOI:** 10.1371/journal.pone.0172800

**Published:** 2017-04-10

**Authors:** Rosalinda Madonna, Francesca Vera Renna, Paola Lanuti, Matteo Perfetti, Marco Marchisio, Carlo Briguori, Gerolama Condorelli, Lamberto Manzoli, Raffaele De Caterina

**Affiliations:** 1 Center of Aging Sciences and Translational Medicine – CESI-MeT, “G. d’Annunzio” University, Chieti, Italy; 2 Institute of Cardiology, Department of Neurosciences, Imaging, and Clinical Sciences, “G. d’Annunzio” University, Chieti, Italy; 3 Center of Aging Sciences and Translational Medicine – CESI.MeT, Chieti, Italy; 4 Department of Medicine and Aging Sciences, “G. d’Annunzio” University, Chieti, Italy; 5 Clinica Mediterranea, Naples, Italy; 6 Department of Molecular Medicine and Medical Biotechnologies "Federico II” University, Naples, Italy; 7 Center of Aging Sciences and Translational Medicine – CESI-MeT, Chieti, Italy; 8 Department of Medicine and Aging Sciences, “G. d’Annunzio” University, Chieti, Italy; 9 Department of Medical Sciences, University of Ferrara, Ferrara, Italy; Regional Healthcare Agency of Abruzzo, Pescara, Italy; Universita degli Studi di Milano, ITALY

## Abstract

**Rationale and objective:**

Endothelial progenitor cells (EPCs) play a role in vascular repair, while circulating endothelial cells (CECs) are biomarkers of vascular damage and regeneration. Statins may promote EPC/CEC mobilization in the peripheral blood. We evaluated whether pre-procedural exposure to different lipid-lowering drugs (statins±ezetimibe) can acutely increase levels/activity of EPCs/CECs in patients with stable coronary artery disease (CAD).

**Methods:**

In a planned sub-analysis of the *Rosuvastatin For* *RE**duction Of* *M**yocardial* *D**amag**E* *During Coronary Angioplast**Y* (REMEDY) trial, 38 patients with stable CAD on chronic low-dose statin therapy were randomized, in a double-blind, placebo-controlled design, into 4 groups before PCI: *i*. placebo (n = 11); *ii*. atorvastatin (80 mg+40 mg, n = 9); *iii*. rosuvastatin (40 mg twice, n = 9); and *iv*. rosuvastatin (5 mg) and ezetimibe (10 mg) twice, (n = 9). At baseline and 24 h after treatment–before PCI–, patients underwent blinded analyses of EPCs [colony forming units-endothelial cells (CFU-ECs), endothelial colony-forming cells (ECFCs) and tubulization activity] and CECs in peripheral blood.

**Results:**

We found no significant treatment effects on parameters investigated such as number of CECs [Median (IQR): *i*. 0(0), *ii*. 4.5(27), *iii*. 1.9(2.3), *iv*. 1.9(2.3)], CFU-ECs [Median (IQR): *i*. 27(11), *ii*. 19(31), *iii*. 47(36), *iv*. 30(98)], and ECFCs [Median (IQR): *i*. 86(84), *ii*. 7(84), *iii*. 8/(42.5), *iv*. 5(2)], as well as tubulization activity [total tubuli (well), Median (IQR): *i*. 19(7), *ii*. 5(4), *iii*. 25(13), *iv*. 15(24)].

**Conclusions:**

In this study, we found no evidence of acute changes in levels or activity of EPCs and CECs after high-dose lipid-lowering therapy in stable CAD patients.

## Introduction

Stem/progenitor cell transplantation or mobilization are emerging as promising new treatments for heart failure and myocardial infarction [1]. Endothelial progenitor cells (EPCs) are rare progenitor cell subsets originated in the bone marrow, which are mobilized upon specific stimulation [2–4], then homing to target tissues where they are involved in endothelial repair or remodeling, as well as in post-natal neo-vasculogenesis [5]. EPCs may contribute to cardiac and vascular repair [6–8]. EPCs also appear to promote cardioprotection and cytoprotection by releasing paracrine factors, such as vascular endothelial growth factor (VEGF), granulocyte-colony stimulating factor (G-CSF), stem cell-derived factor (SDF)-1α and insulin growth factor (IGF)-1 [9, 10].

Among their so called “pleiotropic effects”, independent of low-density lipoprotein (LDL) reduction, statins have been shown to efficiently increase levels of EPCs in patients with coronary artery disease (CAD) [11] and in patients with chronic heart failure [12], and to improve the proliferative capacity of EPCs, in a way similar to VEGF [11, 13, 14]. Such effects have been ascribed to the regulation of stem/EPC gene expression and function [15, 16]. Treatment with statins may also inhibit stem cell apoptosis and increase their proliferation [17]. Statins inhibit 3-hydroxy-3-methylglutaryl coenzyme A (HMG-CoA) reductase, a rate-limiting enzyme catalyzing the conversion of HMG-CoA to mevalonic acid. In addition to improving lipid profile by reducing LDL cholesterol levels, statins reduce the incidence of myocardial infarction after percutaneous coronary intervention (PCI) [18, 19]. In the randomized ARMYDA (Atorvastatin for Reduction of MYocardial Damage during Angioplasty) RECAPTURE trial, reloading with high-dose atorvastatin has been reported to improve clinical outcomes of patients on chronic statin therapy undergoing PCI [20]. Other described acute effects of statins in the setting of PCI [21] include the modulation on endothelial function, inflammation and thrombosis, with mechanisms not completely understood [22]. One hypothesis is that pre-procedural intensive statin treatment activates cardioprotection and vascular repair by stimulating proliferation, mobilization and homing of EPCs, with subsequent augmentation of circulating and cardiovascular tissue-resident EPCs, in a manner independent of cholesterol synthesis [23].

We tested this hypothesis in the setting of a pre-specified subanalysis of patients enrolled in the Rosuvastatin for REduction of Myocardial damagE and systemic inflammation During coronary angioplasty (REMEDY) trial, specifically investigating whether acute statin treatment before PCI is accompanied by (and is therefore possibly mechanistically linked to) an increase in CEC/EPC levels and functional activities.

## Materials and methods

### Study population and design

The present report derives from a pre-defined single-institution (“G. d’Annunzio” University Cardiology Division at Chieti, Italy) substudy of the *Rosuvastatin For* *RE**duction Of* *M**yocardial* *D**amag**E* *During Coronary Angioplast**Y* (REMEDY) clinical trial.

REMEDY (Eudract Number: 2009-013622-17; ClinicalTrials.gov Identifier = NCT02205775) was a prospective, multicenter, double-blind, randomized clinical trial, examining consecutive patients with stable CAD or a previous acute coronary syndrome (ACS) off the acute phase, undergoing elective percutaneous coronary intervention (PCI) with stenting. The design of the study, as well as of its main substudies, have been published [23]. The primary results of the REMEDY trial have not been published and likely will not, because, when less than half the projected patients had been included, the sponsoring company with a unilateral decision decided to interrupt the funding. Therefore, only two centers, Chieti and Naples, continued the mechanistic substudies with their own resources, only relying upon the availability of the randomization treatments still available.

**Fig 1:**
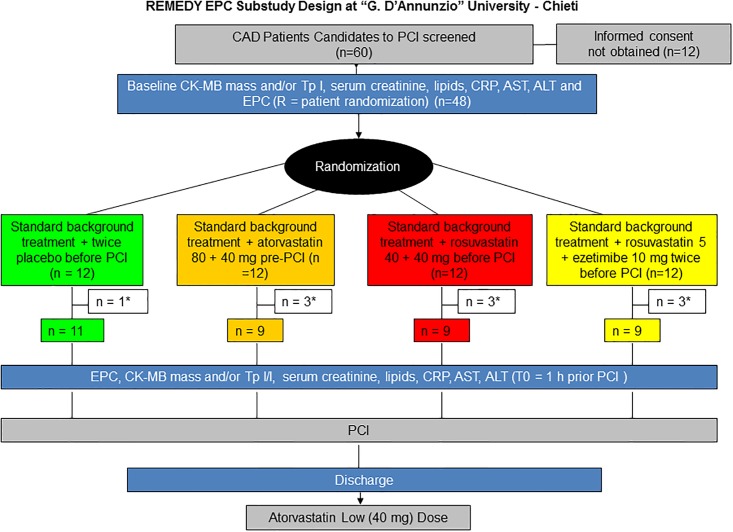
Study design of the REMEDY-EPC early substudy. Coronary artery disease (CAD) patients candidate for an elective percutaneous coronary interventions (n = 60) were screened, Forty-eight of them, providing written informed consent, were enrolled and randomly allocated to 4 treatment strategy groups comprising placebo or 3 lipid-lowering treatments, as illustrated. The final analysis consisted of n = 38 patients due to some missing sampling (indicated by *), as illustrated. Sampling for the identification and quantification of endothelial progenitor cells (EPCs) and circulating endothelial cells (CECs) were performed at the time of randomization (sample R), and 1 hour before the diagnostic angiography and PCI, at time of treatment reload (placebo or lipid-lowering treatments, sample T0).

The design of the present substudy is illustrated in Fig 1. Inclusion criteria, as for the main REMEDY trial [23], were: 1) stable coronary artery disease (CAD) with inducible myocardial ischemia and indication to coronary angiography; or 2) non-ST-segment (NSTE) ACS or STEMI deemed to require an invasive strategy, but with stabilized markers of myocardial necrosis (CK-MB or troponins, with variation <20% in 2 consecutive measurements obtained at ≥6 h time distance before PCI, according to the second universal definition of periprocedural myocardial infarction [24]). Exclusion criteria, as in the main REMEDY trial, were: STEMI or NSTE-ACS with high-risk features warranting emergency coronary angiography: any increase in liver enzymes (aspartate amino transferases/alanine amino transferases) ascribed to liver dysfunction at baseline; left ventricular ejection fraction <30%; renal failure with creatinine >2 mg/dL; history of liver or muscle disease; ongoing treatment with high-dose statins (atorvastatin 80 mg/day or rosuvastatin 40 mg/day); pregnancy and lactation.

Patients were randomized into 4 treatment groups:

1. standard background treatment (performing PCI on the background of standard treatment, without any change of the therapy received, according to local practice), and placebo twice immediately before PCI;
2. standard background treatment plus atorvastatin 80 mg + 40 mg before PCI (same daily dosage as in the ARMYDA study [25–27]);
3. standard background treatment plus rosuvastatin 40 mg twice before PCI;
4. standard background treatment + rosuvastatin 5 mg + 10 mg ezetimibe twice before PCI (dosages expected to be equipotent, in terms of LDL cholesterol reduction, to the rosuvastatin regimen, but testing a largely HMG-CoA reductase inhibition-independent way of reducing LDL cholesterol).

Due to the acute nature of the interventions, no changes in plasma lipids were expected as the result of treatment, and therefore were not sought. No independent measures of treatment intake were therefore obtained. However the intake of the double-blinded study medications was witnessed by the investigators responsible of their administration.

Informed consent was obtained from all patients. This specific substudy, in addition to the main study, was approved by the local Ethics Committee (Full name: “Comitato Etico delle Province di Chieti e Pescara e dell'Universita' degli Studi "G. d'Annunzio" di Chieti-Pescara”). All participants provided their written informed consent to participate in this study. The Ethics Committee approved this consent procedure. Data obtained were managed blindly.

According to the protocol, patients were treated before intervention with aspirin (100 mg/day) and clopidogrel (75 mg/day if on chronic (>3 day) treatment; or given a 300–600 mg loading at least 6 h before the procedure if previously untreated with a P2Y_12_ inhibitor. Procedural success was defined as a residual stenosis <30% diameter. After PCI, aspirin (100 mg/day) was continued indefinitely, whereas clopidogrel (75 mg/day) was administered for at least 1 month (6–12 months in patients treated for ACS or receiving drug-eluting stents). After the intervention, all patients were treated with atorvastatin (40 mg/day), irrespective of the initial randomization assignment.

At the time of randomization and at the time of treatment reload immediately before the diagnostic angiography and PCI, peripheral blood was collected to measure CEC levels and EPC levels and functional activity (see below). In addition, plasma lipids (total cholesterol, high-density lipoprotein (HDL) cholesterol, triglycerides, to derive low-density lipoprotein (LDL) cholesterol according to the Fredrikson formula), were measured before treatment; creatine kinase (CK), CK-MB (mass), troponin-I (mass), and myoglobin were assayed–before and after the acute treatment–with standard centralized laboratory assays. The upper limits of normal (ULN) were defined as the 99th percentile of a normal population with a <10% total imprecision according to the Joint European Society of Cardiology/American College of Cardiology Universal Myocardial Infarction definition [24]. C-reactive protein (CRP) levels were also measured at the same time points. CRP were assayed by the Kriptor ultrasensitive immunofluorescent assay (Brahms, Hennigsdorf, Germany), with a detection limit of 0.06 mg/L.

#### Endpoint definitions

The primary objective of the REMEDY-EPC early substudy was to compare changes of CEC levels and EPC levels and functional activities essential for vasculogenesis (early and late EPC colony formation, proliferation, migration and tube formation) resulting from the acute randomization treatments before PCI.

### Collection and processing of peripheral blood for CEC and EPC levels

To monitor changes in CEC and EPC levels, 12 mL peripheral blood (the minimum amount required to obtained a visible 3-layer stratification for mononuclear cell isolation) were collected into ethylenediaminetetraacetic acid (EDTA)-treated tubes (4 tubes/patient) at the time of randomization (sample R), and at the time of treatment reload (sample T0), this latter performed 1 h before diagnostic angiography and PCI. Blood samples were maintained in EDTA-treated tubes at +4°C, and used within 5 h for mononuclear cell (MNC) isolation and assays of functional activities.

#### CEC/EPC isolation

All culture methods to identify CEC/EPC levels require a preliminary step of mononuclear cell isolation from peripheral blood. Therefore, peripheral blood mononuclear cells were isolated from 12 mL of peripheral blood by gradient centrifugation using Ficoll-Paque PLUS (Amersham). Twelve mL of peripheral blood (in 4 ethylenediaminotetraacetic acid (EDTA)-containing vacutainers [BD Vacutainer Eclipse, 21 G x 1-1/4”, 0.8 x 32 mm), in EDTA (2 mg/mL) tubes (BD K_2_E EDTA, Becton Dickinson Biosciences (BD), San Jose, CA, USA] were mixed with 1 part of phosphate-buffered saline (PBS). An equal volume of Ficoll was placed in a 50 mL Falcon tube, and the blood-PBS mixture was carefully stratified onto Ficoll. The tube was then centrifuged at 400 x g at 20°C for 35 min. Three layers were obtained at the end of centrifugation: a. an upper layer, containing plasma + PBS; b. a middle layer, containing monocytes and lymphocytes; c. a lower layer, containing Ficoll, neutrophils and erythrocytes. The middle layer was gently aspirated and placed in a new 50 mL Falcon tube, to which 25 mL cold PBS were added, and then further centrifuged at 400 x g for 5 min. The upper layer was then snap-frozen at -80°C, in 3 mL aliquots, in cryovials for further biochemical determinations (for adhesion molecules, growth factors, and cytokines, not the subject of this report). The pellet was then resuspended in 30 mL PBS/5% fetal calf serum, centrifuged at 400 x g for 5 min, washed again, then used for colony forming units-endothelial cells (CFU-EC) and the isolation of late outgrowth colonies.

#### CFU-EC isolation and quantification

**Fig 2:**
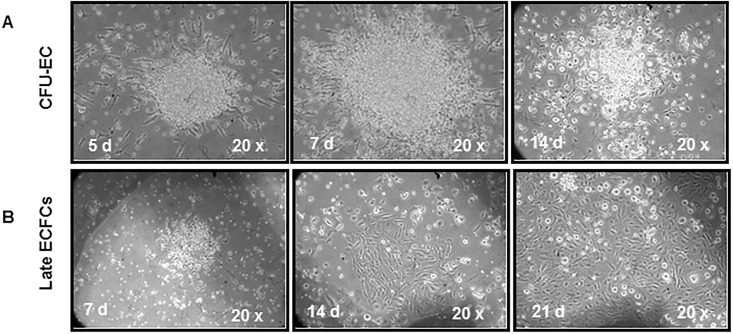
Colony forming units-endothelial cells and late endothelial colony forming cells. Representative images of colony forming units-endothelial cells or CFU-Hill (A) and late endothelial colony forming cells or ECFCs (B) in patients at time of treatment reload.

CFU-EC also known as CFU-Hill [28]) were cultured using the EndoCult^®^ Liquid Medium kit (Stem Cells Inc., Vancouver, Canada), according to the manufacturer’s instructions and as described in detail previously [29]. The cells organize in small clusters of central rounded cells, with radiating spindle-shaped cells that disappear from the 10–14 day on (Fig 2, panel A). At day 5 after plating in fibronectin-coated 24-well plates, clusters were counted in 8 randomly selected high-power (20x magnification) fields.

#### Isolation and quantification of late outgrowth colonies

Late outgrowth colonies, also called endothelial colony forming cells (ECFCs), were isolated as previously described [30]. Briefly, 1 × 10^6^ peripheral blood mononuclear cells were plated onto fibronectin-coated 6-well plates, and cultured in endothelial cell basal medium (EBM)-2 (Cambrex Bio Science Walkersville Inc., Walkersville, MD, USA), with Endothelial Cell Growth Medium (EGM^™^)-2 supplement (Cambrex) for 15 days. The culture medium was changed first on day 4, and then every 2 days. With this culture system, attaching cells rapidly assume an endothelial-like shape, and starting from days 3–6 of culture, proliferate in clusters or small colonies made-up of a central core of rounded cells surrounded by radiating spindle-shaped cells. Starting from days 10–14, cells also organize in large colonies with a cobblestone appearance, which are considered endothelial colonies, and become confluent starting from days 21 (Fig 2 panel B). Clusters were visualized under an inverted microscope every 3 days starting from days 7–14, and counted, at days 14 and 21 after plating, in 8 randomly selected 10x magnification fields.

### Tube formation assay

The tube formation assay was performed on late ECFCs collected at days 21, with a minimal volume of Matrigel in 96-well plates (BD), which allows the formation of both tubules and a vascular network. After detachment from the plates at day 21 with the use of 0.25% trypsin, 1 × 10^4^ late ECFCs were placed on a matrix solution with EGM-2 microvascular (MV) medium, and incubated at 37°C for 16 h. Tube formation was monitored with an inverted phase-contrast microscope (Leica, Wetzlar, Germany), and picture taken by an attached digital output Olympus camera. Tube formation indices (including tube areas, tube length, and tube number) were quantified with the National Institutes of Health (NIH) version 1.49 Image software.

### Multi-color flow cytometry

The first 3 mL of peripheral blood withdrawn were discarded and not used for the flow cytometry analysis to avoid the effects of the venipuncture damage on CEC numbers. Every first mL of the blood sampling was instead used to determine the sample leukocyte count, in order to assess total white cell count and lymphocyte absolute count by using double platform counting.

#### Instrument setting

Once set, the performance, stability, and data reproducibility of the flow cytometer were daily checked in real time using the BD^™^ Cytometer Setup and Tracking (CS&T) Quality Control Module, and further validated by the acquisition of Spherotech 8 Rainbow Beads peaks (Spherotec. Lake Forest, IL, USA), as well as of CS&T bright beads [31]. Afterwards, stabilization of the laser lamp was done for a period of 30 min.

#### Reagents/antibodies panel

**Fig 3:**
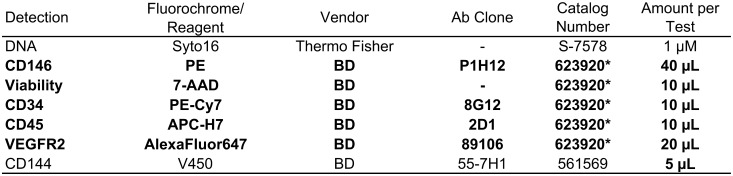
Flow cytometry specificities and reagents. Reagents composing the lyophilized panel are evidenced in bold face. Other reagents added to the basic panel are listed in plain fonts. *Catalog number of the lyophilized combination. Abbreviations: PE = R-phycoerythrin; 7-AAD = 7-AminoActinomycin D; Cy7 = PE-Cyanine; APC-H7 = Allophycocyanin-Hilite^®^7; BD = Becton Dickinson Biosciences (San Jose, CA, USA).

**Fig 4:**
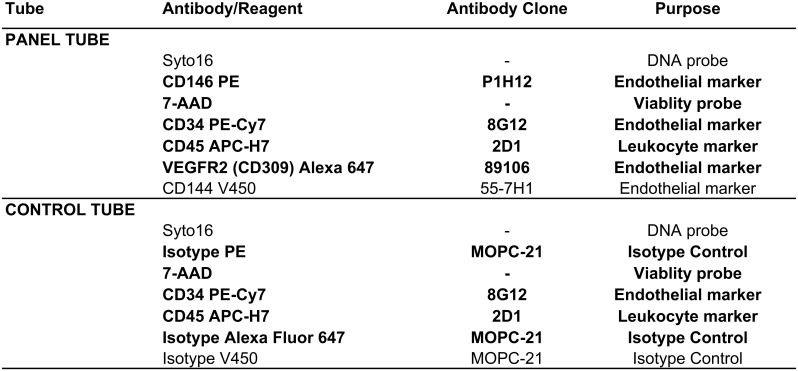
Composition of the flow cytometry panel and control tubes. Reagents composing the lyophilized panel are evidenced in bold face. Other reagents added as liquid drop in to the basic panel are listed in plain fonts. Abbreviations: PE = R-phycoerythrin; 7-AAD = 7-AminoActinomycin D; Cy7 = PE-Cyanine 7; APC = Allophycocyanin = APC; APC-H7 = Allophycocyanin-Hilite^®^7.

CECs were identified as CD34^bright^/CD45^-^/CD144^+^/CD146^+^ events, together with DNA staining and dead cell exclusion, by using an already established panel of reagents [31]. To improve standardization, liquid reagents for the panel and the respective control tube (Figs 3 and 4) were lyophilized as described [31]. A single lot of lyophilized reagent tubes was used for the entire study.

#### Sample staining

For each sample, 20 x 10^6^ leukocytes were processed within 4 h from blood collection, as described [32]. Briefly, the sample volume containing 20 x 10^6^ leukocytes underwent an erythrocyte lysis step through the addition of 45 mL of Pharm Lyse solution (BD) for 15 min at room temperature under gentle agitation, as per the manufacturer’s instructions. Samples were then centrifuged (400 x g for 10 min at room temperature) and washed by adding 2 mL of staining buffer, containing bovine serum albumin (BD). Surface staining was carried out by adding the pellet of each sample to the re-hydrated lyophilized cocktail of reagents. To this, 1 μM Syto-16 (Thermo Fisher Scientific, Waltham, MA, USA) and V450-conjugated anti-CD144 (i.e. conjugated with fluorochrome V450, with 406 nm excitation and 450 nm emission) were added as a liquid drop to each panel tube (Fgures 3 and 4). Samples were incubated in the dark for 30 min at 4°C, washed into 2 mL of staining buffer with bovine serum albumin, and resuspended in 1.5 mL of FACSFlow buffer (BD).

#### Data acquisition

A minimum of 2 x 10^6^ and a maximum of 4 x 10^6^ events/sample with lymph-monocyte morphology were acquired by flow cytometry with a FACSCanto apparatus (BD) at a “medium” flow rate (60 μL/min of sample). A threshold combination was used on the forward scatter (FSC) and the fluorescein isothiocyanate (FITC) (Syto16) channels to get rid of very small and non-nucleated events. The specificity of anti-CD144, anti-CD146 and anti-VEGFR2 bindings were assessed with the use of isotype-matched controls at the same concentration and from the same manufacturers of the respective specific antibodies. Compensations were done using CompBeads (BD) and single-stained fluorescent cells. Carryover between samples was prevented by appropriate instrument cleaning at the end of each sample acquisition.

#### Gating strategy

Events displaying the typical lymph-monocyte morphology were first selected in a forward scatter (FSC) versus side scatter (SSC) plot. Next, dead cells were excluded (on a 7-amino-actinomycin D (7-AAD)/FSC dot plot) on the basis of their positivity to 7-AAD, and nucleated events were gated. The aforementioned 3-gating plots were intersected, and cells resulting from this logical combination characterized by lymph-monocyte morphological features, being also alive and nucleated, were then analyzed for CD45 and CD34 expression on a CD45/CD34 dot plot. The whole CD34^+^ cell compartment was identified, and two subpopulations, displaying different levels of CD34 surface expression, were identified and gated separately. These were CD34^+^ cells, which are CD45dim and represent the hematopoietic stem cell compartment; and a CD34 bright population, which resulted to be CD45 negative (CD45^-^). Both the hematopoietic stem cell (HSC) and the CD34^bright^/CD45^-^ cell populations were then analyzed for the expression of CD144, CD146 and CD309, on CD144/CD34, CD146/CD34 and CD309/CD34 dot plots, and compared with their respective control tube dot plots, containing the isotype controls.

#### Data analysis

Data were centrally collected and analyzed by a single operator by using the FACSuite v1.04 (BD) software. To ensure the correct identification of negative and positive populations, cells were plotted using a dot-plot bi-exponential display. In order to assess non-specific fluorescence, both fluorescence minus one (FMO, a type of control employed in experiments using multiple dyes, in which all labels are included except the one suspected to give spectral overlap) and isotype controls in combination with all the remaining surface reagents present in the panels were used [33, 34].

#### Counting formula

CECs and hematopoietic stem cell numbers were calculated by a dual-platform counting method, and absolute numbers were obtained by using the already reported formula [31].

### Statistical analyses

All parameters of in vitro angiogenesis and clonogenic activity, as well as antigen expression measured before and after treatments were compared with the Wilcoxon matched-pairs signed ranks test to evaluate possibly significant changes. Baseline characteristics among the intervention groups were compared using the Fisher's exact test for categorical variables, and the Wilcoxon signed-rank test for continuous ones. The Kruskal-Wallis test was then used to assess whether the post-pre difference in each parameter differed by treatment group, gender, smoking, history of cardiovascular diseases (CVD), hypercholesterolemia, dyslipidemia, diabetes, hypertension, and diagnosis of stable CAD.

As a secondary analysis, Spearman's correlation coefficients were used to explore the relationships between between couples of post-pre differences for each parameter. Statistical significance was defined as a two-sided p-value <0.05 for all analyses. These were carried out using the Stata 2013 version 13.1 software (Stata Corp., College Station, Texas, USA). A formal sample size calculation to detect differences in the various study arms was not performed, due to the lack of precise data on the topic in the literature. All analyses are therefore to be considered exploratory and underpowered, and p-values are therefore not reported. Patients whose blood sample was not enough for both analysis (flow cytometry and colony assay) were not included in the study and the blood was discarded in such cases.

## Results

### Patient characteristics

Patient recruitment for this study started in November 2011 and was completed in November 2013. A total of 60 patients, candidates for coronary angiography were screened. Of these, 12 patients did not give their consent to the study and were therefore withdrawn from further analysis. For 10 patients, blood samples sufficient for EPC and CEC analyses at both time points were not available. Thus, 38 patients were finally included and analyzed. Their mean age was 71 ± 5 years, and 74% were men. Of these 38 patients, 9 received atorvastatin 80 mg + 80 mg, 9 received rosuvastatin 40 mg + 40 mg, 9 received rosuvastatin 5 mg and ezetimibe 10 mg, and 11 received placebo (Fig 1).

**Fig 5:**
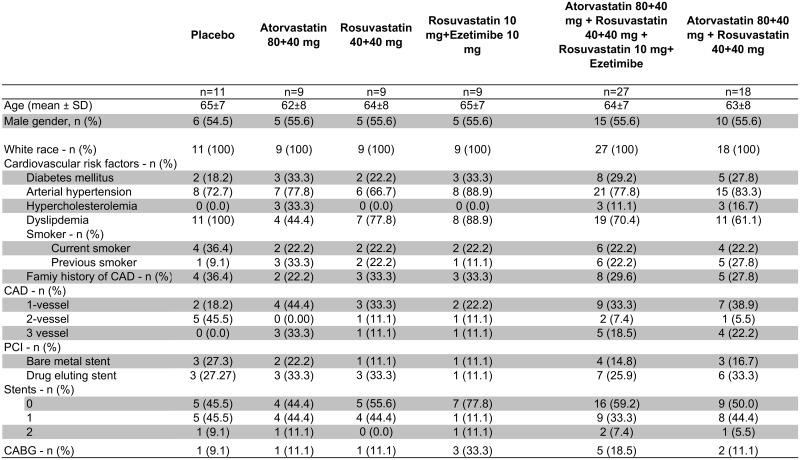
Baseline characteristics of the population studied, divided in the various study groups. Abbreviations: SD = standard deviation; CAD = coronary artery disease; PCI = percutaneous coronary intervention; CABG = coronary artery bypass graft surgery; CAD = coronary artery disease.

Patient characteristics are depicted in Fig 5. 50% of the study population had a previous ACS off the acute phase. There were no statistically significant between-group differences in any of the group characteristics, and no differences were also found between patients who received any lipid-lowering agents, however combined, and those who received placebo.

### Effects of treatments on indices of myocardial damage, and side effects

**Fig 6:**
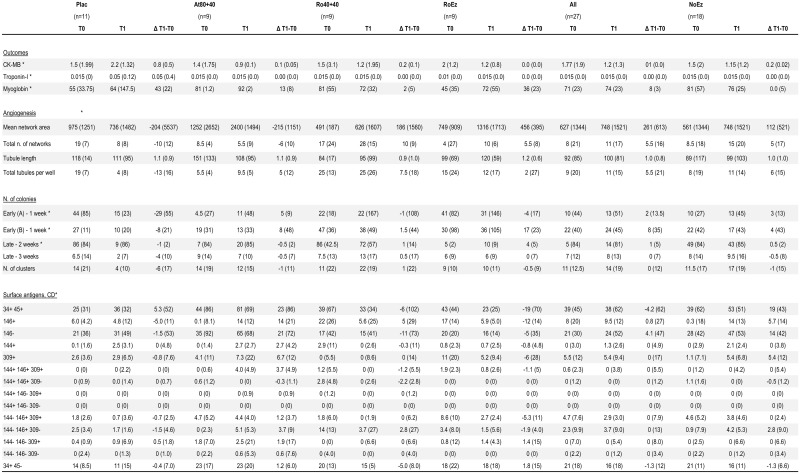
Selected markers of myocardial damage, parameters of angiogenesis and clonogenic activities, and surface antigens before and after treatment. For each parameter, at each time point, numbers represent the median and the interquartile range (this latter in parenthesis). Abbreviations; Plac = placebo; At80+40 = atorvastatin 80 mg + 40 mg; Ro40+40 = rosuvastatin 40 mg + 40 mg; RoEz = rosuvastatin + ezetimibe (these are the treatment groups as defined in Methods); T0, T1 = time 0, time 1, as defined in Methods; Δ T1-T0 = Difference T1-T0; CK-MB = creatine kinase-MB isoform; Early and Late Colonies = as defined in Methods; CD = cluster of differentiation—see Methods for details.

Due to the short course of treatment, no effects on plasma lipids were hypothesized, and not actually investigated. No changes in any of the other laboratory parameters assessed to monitor myocardial damage, including myoglobin, CK-MB or troponin-I were found (Fig 6).

### Effects of treatments on EPCs and CECs

In patients treated with ezetimibe and/or high-dose statins, the change of mean number of EPCs-CFUs/plate and EPCs-ECFCs/plate (Fig 6), as well as in any parameter of EPC-ECFC tubulization activity (Fig 6), were all non-significant compared with placebo (for all, p>0.05). Changes in EPC-CFU-EC and EPCs-ECFC levels, and in any parameter of tubulization activity of EPCs-ECFCs as the result of treatments (after treatment–before treatment = Δ) were non-significant (p>0.05). In patients treated with ezetimibe and/or high-dose statins there was a trend towards an increase of EPC level after active treatments as compared with placebo, but this was not statistically significant. This trend was however only observed in patients treated with high-dose rosuvastatin or the rosuvastatin+ezetimibe combination, and not in the atorvastatin group.

For flow cytometry analyses, a gating strategy was used, by which events displaying any lymph-monocyte morphology, alive and nucleated, were firstly gated, while dead cells and non-nucleated events were excluded in order to remove non-specific signals. Alive and nucleated lymph-monocyte cells were then analyzed for their expression of CD34 and CD45. Two different levels of CD34 surface expression allowed the identification of distinct CD34^+^ cell subpopulations. A subset of CD34^+^/CD45^dim^ cells, matching the antigen profile of HSC, and a more restricted subpopulation of cells expressing bright levels of CD34 and resulting to be CD45 negative, were detected. Subsequently, cells staining positive or bright for CD34 and dim or negative for CD45 were analyzed for the endothelial cell markers CD144, 146 and CD309. We found no significant changes as the result of treatments between the absolute numbers of all CD34^+^/CD45^-^ events (Fig 6) or CECs (CD34^bright^/CD45^-^/CD144^+^/CD146^+^) (Fig 6), or HSC (Fig 6) in the ezetimibe and/or high-dose statin treatments, and no significant differences vs the placebo group. Furthermore, all subsets of CD34^+^/CD45^-^ cells, negative for CD144, CD146 and CD309 (Fig 6), or resulting from the different combinations of CD144, CD146 and CD309 (Fig 6) did not show any significant differences in any of the active treatment groups, or in the placebo group.

**Fig 7:**
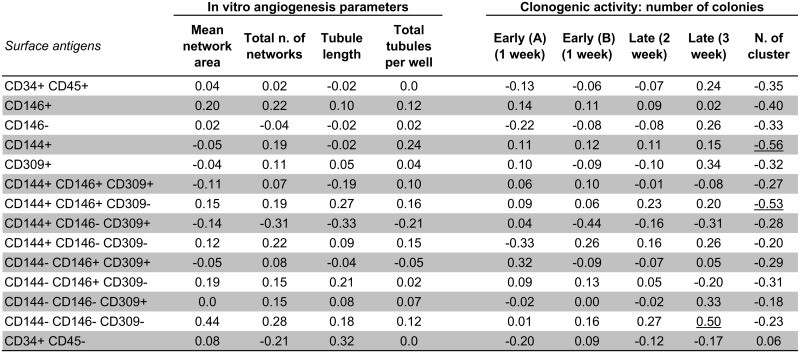
Correlation analysis (Spearman rho) of the differences (Δ = after minus before treatments) in angiogenesis and clonogenic activities (independent variable), and the differences (Δ = after minus before treatments) in surface antigens. Abbreviations; AU = Arbitrary Units; Early (A) and early (B) = Hill colony analysis performed by two independent investigators. Only the underlined correlation coefficients were significant. Treaments = atorvastatin 80 mg + 80 mg, rosuvastatin 40 mg + 40 mg, rosuvastatin 10 mg + ezetimibe 10 mg.

We also found overall no correlation between differences (Δ = after minus before treatments) of EPC numbers and activity (according to cell culture method) and differences (Δ = after minus before treatments) of plasma levels of CECs according to flow cytometry methods here used, with the exception of 3 (out of 126) correlation coefficients (Fig 7).

## Discussion

Contrary to the study hypothesis and to previous findings about an acute increase of EPCs/CECs after high-dose lipid lowering treatments, we here found no evidence of any significant change in either the number or the functional activity of such cells after an acute lipid-lowering treatment. These results were obtained adopting a number of complementary techniques for the identification of circulating progenitors, and in the unprecedented setting of a double-blind, randomized, placebo-controlled study.

Since the acute pre-procedural administration of statins has been associated with cardio- and renal protection, including reduced release of myocardial necrosis markers [22] and reduced incidence of contrast media-induced acute kidney injury [35], the hypothesis that some of these effects may be contributed by an acute mobilization of circulating progenitors had attracted considerable interest. Nevertheless, results from the studies of EPCs and statins in the context of periprocedural myocardial injury, such as during and after PCI, have yielded quite equivocal or discordant results [36]. This may reflect on the one hand the quality of the experimental setting in which such hypotheses were tested; and, on the other hand, the varying manner by which EPCs had been quantified.

There are two possible ways to identify and characterize EPCs: by flow cytometry, looking at cell surface antigenic markers; and by cell culture methods, looking at their functional activities (reviewed in [37]). Currently, 3 main cell culture methods are used to identify 3 types of EPCs with different functions and angiogenic potential: late outgrowth colonies, also known as ECFCs, which are considered as genuine or true EPCs [30]; colony-forming unit (CFU)-Hill cells [28]; and early outgrowth EPCs, also known as circulating angiogenic cells (CACs) [38] (reviewed in [37]). ECFCs display a high proliferative potential, express endothelial antigens, form capillary-like structures when plated on Matrigel, but also blood vessels when suspended in collagen or Matrigel scaffolds and implanted in immunodeficient mice (reviewed in [37]). CFU-Hill cells and CACs display a low proliferative potential, but a great potential to secrete an array of angiogenic cytokines. CFU-Hill cells and CACs are positive for CD45, CD34, CD31 and KDR, and have the capacity to uptake uptake acetylated LDL labeled with 1,1’-dioctadecyl-3, 3, 3’, 3’-tetramethyl-indocarbocyanine perchlorate (DiDLDL)) [37]. As to the identification and enumeration of EPCs by flow cytometry, EPC antigenic profile continues to be controversial [33, 39], and no consensus guidelines have been agreed upon so far [39, 40]. Therefore, in the present study we decided to analyze CECs by flow cytometry, for which there is at least some agreement in identifying such cells as CD34^bright^/CD45^-^/CD144^+^/CD146^+^ events, together with DNA staining and dead cell exclusion [33, 41]. CECs are characterized by mature endothelial features and detach from the vessel walls following vascular damage or because of their physiological turnover [42, 43]. Their levels in peripheral blood have been reported as decreased after statin treatment [44]. Similarly as for EPCs, CEC characterization and enumeration in the bloodstream has been proposed as a potential biomarker of the response to treatment in several clinical conditions involving vascular damage, regeneration and growth [4, 41, 45–47]. Likewise, preclinical and clinical studies have suggested that EPCs play an essential role in neovascularization, and are biomarkers of atherosclerosis, inversely related to the presence and progression of the disease (reviewed in [37]).The balance of CEC and EPC levels would reflects the endothelial health status [43, 48].

We examined whether the reported beneficial effect of statins in reducing periprocedural myocardial injury is due to their effects on EPCs. We also addressed the important question of whether this is a class effect or, on the contrary, differences exist between rosuvastatin, atorvastatin and the low-dose rosuvastatin/ezetimibe combination. These 3 treatments were selected because achieving similar effects on LDL lowering on chronic treatment. While the first two treatments determine a robust LDL lowering through a substantial inhibition of HMGCoA reductase [49], the third does so by combining a relatively milder HMGCoA reductase inhibition with an interference in LDL intestinal absorption with ezetimibe [50, 51]. The setting of the REMEDY trial, by which these 3 active treatments were compared with placebo in a double-blind double-dummy design, overcomes several of the methodological biases attributable to less rigorous study design, as adopted in virtually all previous studies on this topic. This report addresses the possible impact of tested regimens on early EPC levels and functional activity; another report will deal with longer-term (3-months) effects [23].

Here we prospectively investigated whether the preprocedural exposure to different lipid-lowering drugs (statins and/or ezetimibe) or placebo can acutely (within 24 h) modify levels of CECs and levels and functional activity of EPCs, with potential differences between treatments. While it is generally recognized that statin administration to patients may increase levels of EPCs, identified by varying combinations of surface antigens, in the absence of accepted guidelines [39, 40], the time- and dose-response course of such effects by different lipid-lowering agents had not been previously characterized. Our study is therefore the first attempt to assess functional and antigenic levels of EPCs after the acute administration of various lipid-lowering agents, prospectively administered with random allocation and in a double-blind design. In addition, this is the first study attempting to enumerate CECs and any subsets of CD34^+^/CD45^-^ cells, deriving from several combination of endothelial markers (CD144, CD146 and CD309), by flow cytometry in the peripheral blood of CAD patients.

Our data show that the acute (within 24 h) preprocedural administration of different lipid-lowering drugs, at higher doses compared with the usual-doses administered before an elective PCI, is not associated with any evidence of EPC mobilization and/or of any release of CACs from the vessel wall in the peripheral blood, without any differences between either treatments and placebo or among the various treatment here tested. Recently, CECs have been found to define a population of mature endothelial cells, that detach from the vessel walls, following vascular damage or its physiological turnover, and become circulating cells [42, 43]. However, data about the acute effects of statin treatment and PCI on EPCs are debatable, reflecting the heterogeneity of methods used for the evaluation of EPCs and the great variability in statin treatment timing and dosage [36]. Vasa et al. [11] demonstrated, in a series of 15 patients with stable CAD, that a therapy with atorvastatin 40 mg/day increased the number and functional activity of EPCs after 1 week of treatment. More recently, it has been suggested that the effect on EPC mobilization might be dose-dependent: among 100 patients with ischemic heart disease; here treatment with atorvastatin 40 mg/day was associated with a higher EPC level than a regimen of atorvastatin 10 mg/day [52]. At the same time, other groups have shown that chronic statin therapy can be associated with a reduced–rather than increased–EPC number and function. So, Hristov et al. found that a chronic (3-month) statin treatment was associated with a reduction in the number of circulating EPCs. The dose of continuous statin therapy inversely correlated with EPC number, and patients treated with a 40 mg/day statin (simvastatin or atorvastatin) showed a reduction in EPC number. Interestingly, statin treatment did not affect the functional properties of EPCs tested by the CFU-Hill Assay [53]. Conversely, Deschaseaux et al. found that the number of EPC colonies after 5 days of culture was lower in patients on chronic statin treatment (here with 40 mg/day of pravastatin or simvastatin for at least 4 weeks) compared with those without statin therapy, while long-term statin treatment would preserve late EPC colonies [54]. Recently, a systematic review of preclinical and clinical studies on statin and EPCs [55] reported two clinical studies examining the effect of statin therapy on EPC levels in CAD patients [56, 57]. One study used atorvastatin and the other used rosuvastatin. One study showed an increase in circulating levels of EPCs after 5 days of statin therapy and the other reported greater numbers of CFU-Hill after 6 months of treatment. However, the non-randomized nature of most of these studies and the lack of standard dosing certainly justifies uncertainty in drawing firm conclusions. Data from randomized studies of both early and long-term effects of statin therapy on EPCs are therefore clearly warranted. Our report tries to fill-in such knowledge gap with an appropriate study design and multiple methods to detect and quantify EPCs and CACs. We found no evidence of any acute effect of lipid-lowering therapies on any of the parameters here investigated. We also report no correlations of antigenic vs functional parameters for EPC/CEC assessment (with only 3 out of 126 correlation coefficients turning out to be “statistically significant”, but with no adjustment for multiple testing, which we therefore attribute to the play of chance), casting doubts on the possibility of using such parameters interchangeably in future studies.

### Study limitations

We recognize limitations of our study. Given the relatively small patient population, we cannot rule out the occurrence of a Type 2 statistical error. However, the combination of high statin dose (atorvastatin and rosuvastatin) does not here provide a signal for efficacy, nor such a signal appears even combining the 3 active treatment groups or the similarly acting, atorvastatin and rosuvastatin, with similar potency.

As a second limitation, no data are here available about the EPC behavior in a prolonged follow-up. This was beyond the specific objectives of the present REMEDY-EPC early substudy, but will be the subject of an independent report (the REMEDY-EPC late substudy), as also obtained in a different patient population and in another research center, within the same overall REMEDY study.

Because of the short time of the observation, no independent effects of any of the acute treatment administered could be demonstrated. We did not measure changes in lipid parameters or CRP, clearly shown upon longer treatments [58], as such changes are not expected to occur so acutely. The occurrence of acute administration was however witnessed by the investigators involved, and we can reasonably exclude issues of non-compliance. We did not find any effect of treatments on indices of myocardial damage, suggesting at least no harm from such treatments. The main aim of this pilot study was to provide descriptive data that can be valuable in designing future trials

## Conclusions

Our data, the first obtained after randomization of patients to multiple types of lipid-lowering drugs and at different dosage, and assessing CEC and EPC levels and functional activity, argue for the absence of any mechanistic link between any otherwise observed clinical outcome (observed in other studies) and the effects of early statin therapy on EPCs/CACs in patients with CAD. The absence of any increase in EPC levels and function, whathever the type and dosage of lipid-lowering agent used, appears to reinforce the concept that any beneficial effect of early therapy with lipid-lowering drugs on periprocedural myocardial injury at PCI would be concomitant–and therefore also possibly linked–with the lowering of LDL cholesterol levels, which would require a longer time course, rather than to pleiotropic effects, at least those due to the mobilization of EPCs. This is in line with our findings explaining the effects of statins on stroke largely as the result of LDL cholesterol lowering, in a separate analysis of lipid-lowering clinical trials [59, 60].
